# No Role for Mast Cells in Obesity-Related Metabolic Dysregulation

**DOI:** 10.3389/fimmu.2016.00524

**Published:** 2016-11-24

**Authors:** Jindřich Chmelař, Antonios Chatzigeorgiou, Kyoung-Jin Chung, Marta Prucnal, David Voehringer, Axel Roers, Triantafyllos Chavakis

**Affiliations:** ^1^Department of Clinical Pathobiochemistry, Medical Faculty, Technische Universität Dresden, Dresden, Germany; ^2^Faculty of Science, University of South Bohemia in České Budějovice, České Budějovice, Czech Republic; ^3^Medical Faculty, Institute of Clinical Chemistry and Laboratory Medicine, Technische Universität Dresden, Dresden, Germany; ^4^Paul Langerhans Institute Dresden of the Helmholtz Center Munich at University Hospital and Faculty of Medicine, TU Dresden, Dresden, Germany; ^5^German Center for Diabetes Research (DZD e.V.), Neuherberg, Germany; ^6^Department of Infection Biology, Universitätsklinikum Erlangen at the Friedrich-Alexander Universität Erlangen-Nürnberg (FAU), Erlangen, Germany; ^7^Institute for Immunology, Technische Universität Dresden, Dresden, Germany

**Keywords:** mast cell deficiency, diet-induced obesity, metabolic dysregulation, insulin resistance, glucose tolerance

## Abstract

Obesity-related adipose tissue (AT) inflammation that promotes metabolic dysregulation is associated with increased AT mast cell numbers. Mast cells are potent inducers of inflammatory responses and could potentially contribute to obesity-induced AT inflammation and metabolic dysregulation. Conflicting findings were reported on obesity-related metabolic dysfunction in mast cell-deficient mice, thus creating a controversy that has not been resolved to date. Whereas traditional *Kit* hypomorphic mast cell-deficient strains featured reduced diet-induced obesity and diabetes, a *Kit*-independent model of mast cell deficiency, Cpa3^Cre/+^ mice, displayed no alterations in obesity and insulin sensitivity. Herein, we analyzed diet-induced obesity in *Mcpt5-Cre R-DTA* mice, in which the lack of mast cells is caused by a principle different from mast cell deficiency in Cpa3^Cre/+^ mice or *Kit* mutations. We observed no difference between mast cell-deficient and -proficient mice in diet-induced obesity with regards to weight gain, glucose tolerance, insulin resistance, metabolic parameters, hepatic steatosis, and AT or liver inflammation. We conclude that mast cells play no essential role in obesity and related pathologies.

## Introduction

Inflammation has emerged as an important player in the pathogenesis of obesity-associated metabolic dysfunction and the development of insulin resistance and type 2 diabetes (1–3). In the course of obesity, inflammatory cells, including macrophages (Mφs) and lymphocytes, accumulate in the adipose tissue (AT) and the liver (3, 4). A switch toward the pro-inflammatory (M1) state of Mφs and increased levels of pro-inflammatory mediators, such as TNF, in the obese AT substantially contribute to insulin resistance of adipocytes (1, 5). Therefore, identifying the exact mechanisms and cellular and molecular players governing this immune–adipose crosstalk in obesity is of particular importance.

Mast cells are tissue-resident hematopoietic cells that potently induce inflammatory responses upon ligation of their surface IgE (6). Release of pro-inflammatory mediators by mast cells can be also triggered *via* different pattern recognition receptors on mast cells (7). Therefore, different pro-inflammatory actions ascribed to mast cells in the context of homeostatic or pathogenic immunity that derived from experiments with *Kit* hypomorphic mast cell-deficient mouse strains over the past two decades appeared very plausible (8–10). However, several key findings in *Kit* mutant mast cell-deficient models were not reproduced in novel mouse strains, in which mast cell deficiency was based on principles that were distinct from compromised *Kit* expression. This has led to the assumption that several of the broad actions attributed to mast cells resulting from experiments with *Kit* mutant mast cell-deficient mice may be actually due to disrupted *Kit* function and the complex alterations of the immune system in these strains, rather than mast cell deficiency itself (11). Therefore, the roles mast cells play in the immune system and different pathologies are still unclear.

Few mast cells are found in healthy AT. However, their numbers increase in obesity-related AT inflammation (12–15), which has led to the obvious question whether these cells contribute to obesity-related metabolic dysregulation. *Kit* mutant mast cell-deficient mice of the *Kit^W/W-v^* and the *Kit^W-sh/W-sh^* strains feature improved metabolic parameters upon hypercaloric challenge, including improved insulin sensitivity and glucose tolerance (12). These data raised hopes that metabolic disease might be amenable to therapy targeting mast cells. However, the protection from metabolic dysregulation characterizing the *Kit* hypomorphic mast cell-deficient mouse strains was not observed in a recent study using the novel *Cpa3^Cre/+^* mouse line that lacks mast cells, but expresses normal levels of functional *Kit* (16). In the latter model, in which all mast cells are deleted by genotoxic effects of Cre recombinase expressed at high levels under the control of the carboxypeptidase A promoter (11, 17), no effect of mast cell-deficiency on obesity-associated weight gain, insulin resistance, and AT inflammation was observed (16). The same article demonstrated that the absence of *Kit* itself protected from obesity (16). The controversy was fueled by a recent study based on experiments in *Kit^W-sh/W-sh^* mice, proposing that leptin may regulate the inflammatory phenotype of mast cells, which in turn modulate obesity-related AT inflammation (18).

These controversial findings prompted us to analyze, here, diet-induced obesity in a third independent mouse model of mast cell deficiency, in which the absence of mast cells is caused by a principle different from *Kit* hypomorphic alleles and also from the genotoxic loss of mast cells in Cpa3^Cre/+^ mice (19, 20). The purpose of our study was, therefore, to shed more light onto the controversy regarding the role of mast cells in the development of obesity and related metabolic dysregulation. Our findings unequivocally demonstrate that mast cells do not contribute to obesity-related inflammation and metabolic dysregulation.

## Materials and Methods

### Animals

The *Mcpt5-Cre^+^R-DTA^+^* mouse line was established as described previously (20). Mast cell-deficient (*Mcpt5-Cre^+^R-DTA^+^, n* = 8) and -proficient (*Cre-negative R-DTA^+^, n* = 10) littermate male mice were subjected to a high-fat diet (HFD, 60% calories from fat) (Research Diets, New Brunswick, NJ, USA) for a time period of 21 weeks. Body weight was recorded on a weekly basis. At week 10 of the HFD feeding, metabolic performance was measured in a subgroup of mice using comprehensive laboratory animal monitoring system (CLAMS). Mice were placed in metabolic cages (PhenoMaster; TSE Systems, Bad Homburg, Germany) with free access to water and food and 12/12 h light/dark cycle for three consecutive days and nights. Volume of oxygen consumption (VO_2_) and carbon dioxide production (VCO_2_) were determined automatically every 20 min as well as water consumption and food intake. The respiratory exchange ratio (RER) was calculated as VCO_2_/VO_2_. Motility was recorded by an infrared sensor and measured every 20 min as number of events per period (20 min); averages were calculated for day and night periods. Data were normalized with respect to body weight using analysis of covariance (ANCOVA). Cholesterol and triglycerides levels were measured in blood after 16 h of starvation with AccuTrend (Roche, Mannheim, Germany). Glucose tolerance test (GTT) and insulin tolerance test (ITT) were performed as previously described (21, 22). GTT was performed after 16 h of starvation after 18 weeks on HFD by injecting glucose (1 g/kg of bodyweight) i.p. and by measuring glucose in blood with glucometer (Accu-Chek, Roche, Mannheim, Germany) at different time points, as shown in the figures. ITT was performed after 6 h of starvation after 19–20 weeks on HFD by injecting insulin 1.5 IU/kg of bodyweight i.p. and glucose was measured at different time points, as shown in the figures. After euthanizing mice, sera, subcutaneous and gonadal AT (sAT and gAT), liver, pancreas, and muscle (quadriceps) were collected and used either directly or stored at −80°C for further analysis. Animal experiments were approved by the Landesdirektion Sachsen, Germany.

### Analysis of AT Inflammation

Stromal vascular fraction (SVF) was isolated as described previously (23, 24). Briefly, sAT and gAT were isolated and immediately minced and digested with type I collagenase (Gibco, Darmstadt, Germany). Digested AT was filtered through 100 μm cell strainer (BD, Heidelberg, Germany), and the adipocyte fraction was removed by centrifugation. SVF cells in the pellet were washed and resuspended in FACS buffer (PBS, 0.1%BSA, 0.1%NaN_3_) and analyzed for the content of inflammatory and immune cells by using flow cytometry (FACS Canto II, BD Bioscience). Mφs were detected as CD11b^+^ F4/80^+^; M1 Mφs were detected as CD11b^+^ F4/80^+^ CD11c^+^ cells. Total T cells (CD3^+^) were further divided into T helper (CD3^+^ CD4^+^) and cytotoxic T cells (CD3^+^ CD8^+^). Fc receptors on leukocytes were blocked by anti-CD16/CD32 antibody (BD, Heidelberg, Germany). The following fluorescently labeled antibodies were used: CD11b-APC, CD11c-PE (BD, Heidelberg, Germany), F4/80-Alexa-fluor 488 (eBioscience, Frankfurt, Germany), CD8a-APC, CD4-FITC, CD3e-PE (Miltenyi Biotec GmbH, Bergisch Gladbach, Germany).

### Plasma and Serum Analysis

Plasma was obtained from heparinized blood from mice that were starved for 16 h. Insulin was measured in plasma by using insulin immunoassay (Crystal Chem, Cologne, Germany). After killing mice, the serum was collected and analyzed for adiponectin by using an immunoassay (R&D Systems, Wiesbaden-Nordenstadt, Germany).

### Quantitative Real-Time PCR

Total RNA was isolated from AT and liver by using phenol/chlorophorm extraction (TRI Reagent, MRC, Cincinnati, OH, USA). The expression of inflammatory (IL-1β, IL-6, IL-10, TNF, MCP-1, and F4/80) and metabolic (PPARγ, Srebp1c, LPK, GK, G6P, FATP2, CD36, MTTP and Glut-2) genes (for detailed description see Mast Cell Deficiency Does Not Affect Obesity-Related Liver Steatosis and Inflammation section in Results) was analyzed using SsoFast EvaGreen Supermix (BioRad) on real-time PCR cycler CFX384 (BioRad, Munich, Germany). The expression was normalized to Cre- (mast cell proficient) group by using 18S RNA as a reference and quantified with ΔΔCt method (25). Primers used in the study: mTNF-a_F – AGCCCCCAGTCTGTATCCTTCT, mTNF-a_R – AAGCCCATTTGAGTCCTTGATG, mIL-10_F – TAAGGCTGGCCACACTTGAGA, mIL-10_R – AGCTGCTGCAGGAATGATCA, mF4/80_F – TCAAGGCCATTGCCCAGAT, mF4/80_R – TCCCGTACCTGACGGTTGAG, mIL-1β_F – ATCCCAAGCAATACCCAAAG, mIL-1β_R – GTGCTGATGTACCAGTTGGG, mMCP-1_F – GCATCTGCCCTAAGGTCTTC, mMCP-1_R – AAGTGCTTGAGGTGGTTGTG, mPPARg_F – GAGTGTGACGACAAGATTTG, mPPARg_R – GGTGGGCCAGAATGGCATCT, Srebp1c_F – GATCAAAGAGGAGCCAGTGC, Srebp1c_R – TAGATGGTGGCTGCTGAGTG, mGK_F – TGCGGAGATGCTCTTTGACT, mGK_R – TCTCGGAGAAGTCCCACGAT, mLPK_F – CTTGCTCTACCGTGAGCCTC, mLPK_R – ACCACAATCACCAGATCACC, mG6Pase_F – TGGAGTCTTGTCAGGCATTG, mG6Pase_R – TCCAAAGTCCACAGGAGGTC, mFATP2_F – CTACGCATCCACTGAAGGCA, mFATP2_R – AGTCCAACCTCACCTTTGGG, mCD36_F – AGGTCTATCTACGCTGTGTTC, mCD36_R – ATGGTTGTCTGGATTCTGGAG, Mttp_F – CACTCAGGCAATTCGAGACA, Mttp_R – TCTGGCTGAGGTGGGAATAC, mGlut2_F – ATTCGCCTGGATGAGTTACG, mGlut2_R – CCAGCGAAGAGGAAGAACAC, m18S rRNA_F – GTTCCGACCATAAACGATGCC, and m18S rRNA_R – TGGTGGTGCCCTTCCGTCAAT.

### Histology and Histochemistry

Paraffin stocks were prepared from gAT and liver, 5 μm thick cuts were transferred onto slides and were stained with Mayer’s hematoxylin (SAV, Flintsbach a. Inn, Germany) and counterstained with 1% eosin (Seipt, Germany) for the analysis of adipocyte size and liver steatosis. Mast cell accumulation in gAT was detected by using Giemsa staining (Sigma-Aldrich, Munich, Germany). Steatosis, lobular inflammation, and hepatocellular ballooning were evaluated according to previously published criteria, following the NASH-CRN Committee scoring system (26). Steatosis and inflammation were scored using a 0–3 scale, ballooning by using a 0–2 scale. The NAFLD activity score (NAS) was defined as the sum of steatosis, lobular inflammation, and ballooning, thus ranging from 0 to 8. A microscope coupled to a computerized system (Zeiss, Oberkochen, Germany) and equipped with the AxioVision Rel. 4.8 software (Carl-Zeiss MicroImaging GmbH, Jena, Germany) was used.

### Statistical Analysis

Student’s *t*-test was used for statistical analysis of most experiments, non-parametrical Mann–Whitney *U* test was used for quantitative Real-Time PCR (qPCR) evaluation and ANCOVA, with respect to mouse bodyweight, was used for analysis of data from metabolic cages. All data are expressed as means ± SEM; the level of significance was set at *p* < 0.05.

## Results

### No Difference in Metabolic Parameters between Mast Cell-Deficient and -Proficient Mice

**Figure 1:**
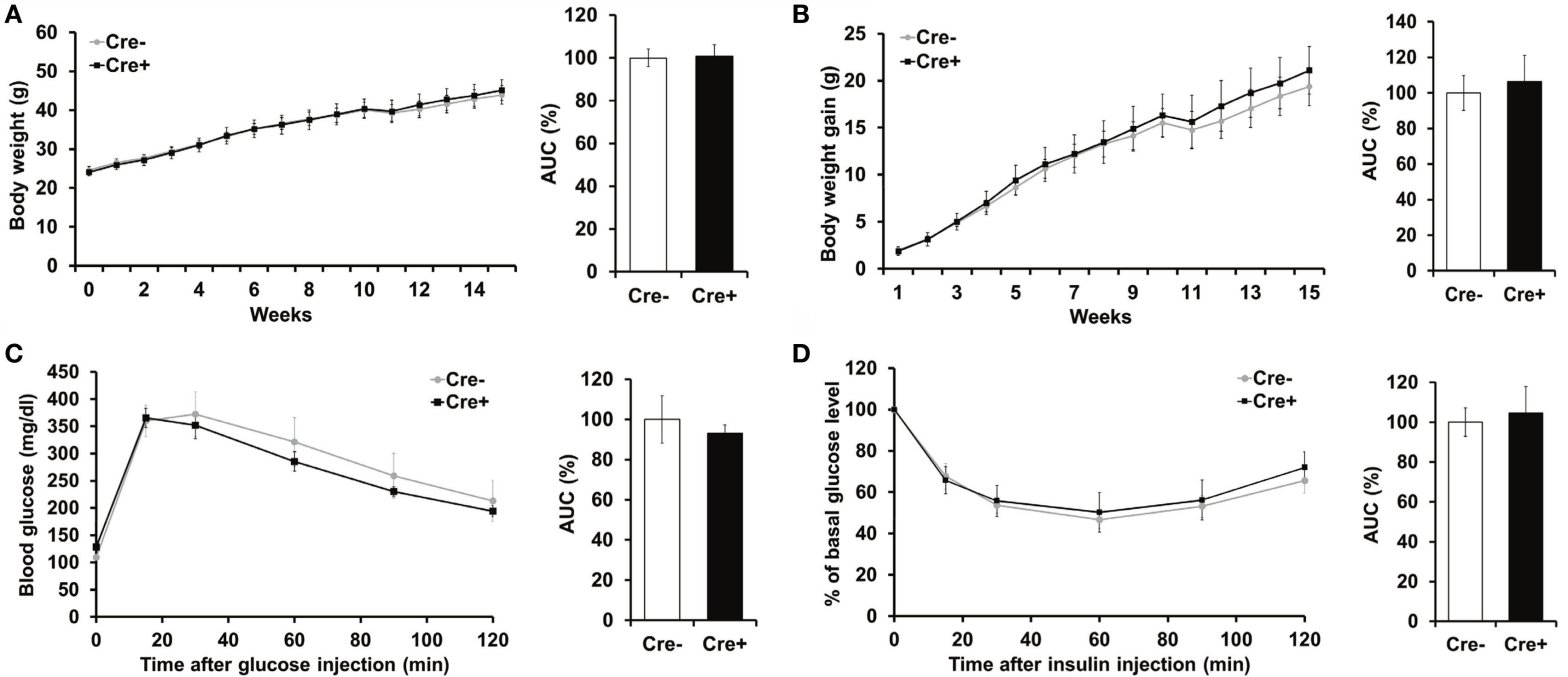
**Mast cell deficiency in the *Kit*-independent *Mcpt5-Cre R-DTA* model does not affect body weight, glucose tolerance, and insulin resistance in diet-induced obesity**. **(A,B)** Mast cell-deficient *Mcpt5-Cre^+^R-DTA^+^* and mast cell-proficient *Cre-negative R-DTA^+^* littermate control male mice were fed a HFD and weight **(A)** and weight gain **(B)** were recorded. **(C,D)** Glucose tolerance test **(C)** and insulin tolerance test **(D)** were performed in mast cell-deficient *Mcpt5-Cre^+^R-DTA^+^* and mast cell-proficient *Cre-negative R-DTA^+^* littermate mice subjected to diet-induced obesity. In **(D)**, data are presented as percentage of initial glucose level. Data and corresponding area under curve (AUC) analysis are shown. AUC data are presented as % of control; the Cre-negative group was set as the 100% control. AUC data were tested for statistical significance with Student’s *t*-test. Data are expressed as means ± SEM (*n* = 8–10 mice).

**Figure 2:**
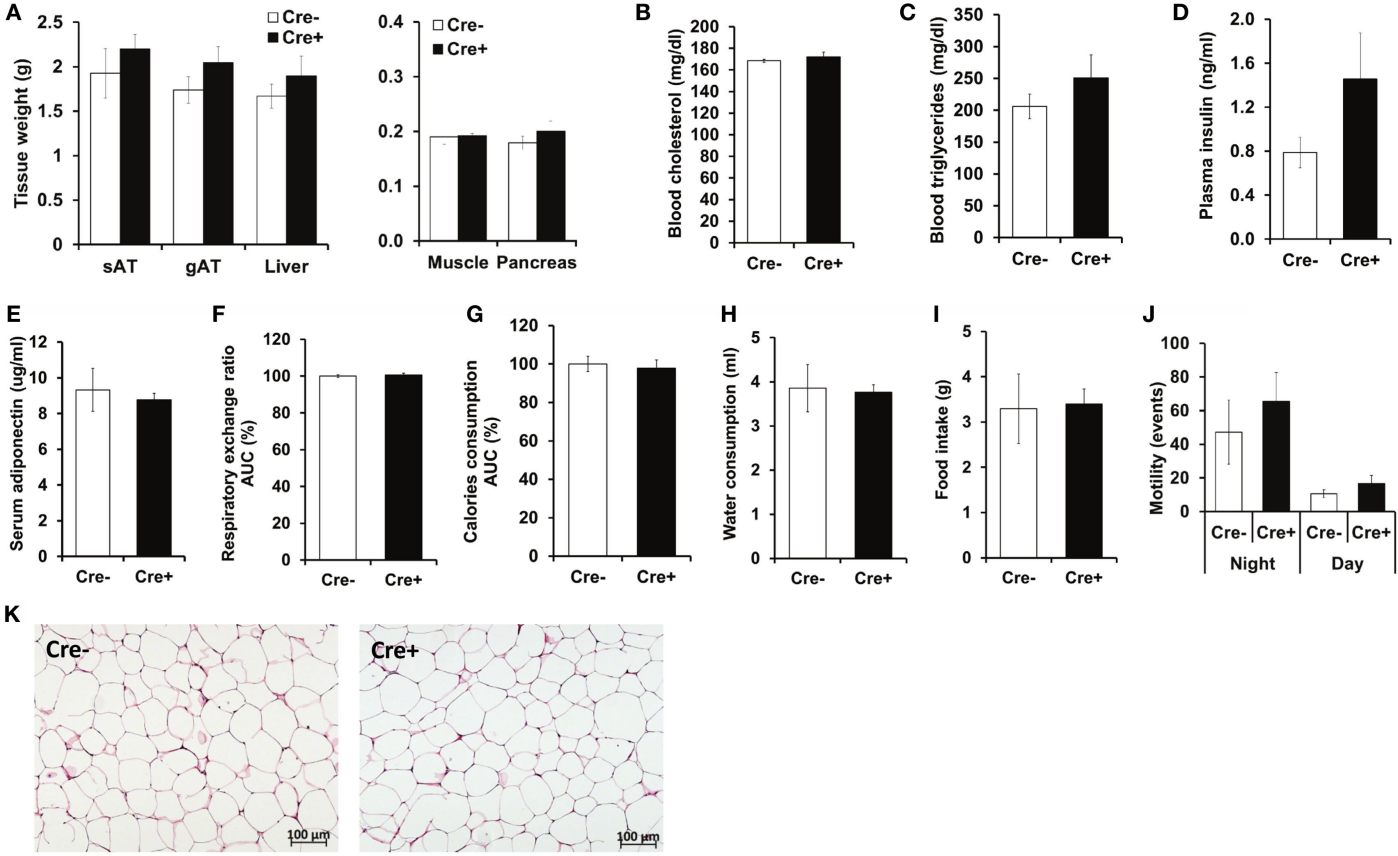
**No metabolic differences between obese mast cell-deficient and -proficient mice**. **(A)** After 21 weeks on HFD, mice were euthanized and dissected. Tissues were collected, and their weights were recorded. sAT, subcutaneous AT; gAT, gonadal AT. **(B,C)** Levels of cholesterol and triglycerides in blood were measured after 16 h of starvation in obese mast cell-deficient and -proficient mice. **(D)** Insulin in the plasma from obese mast cell-deficient and -proficient mice was measured after 16 h of starvation. **(E)** Adiponectin in serum from obese mast cell-deficient and -proficient mice was measured. **(F–J)** Metabolic performance of obese mast cell-deficient and -proficient mice was analyzed by using comprehensive laboratory animal monitoring system (CLAMS) for three consecutive days and nights. **(F)** Respiratory exchange ratio (RER); data are shown as area under curve (AUC) of the experimental period (3 days and 3 nights together). AUC data are presented as % of control; the Cre-negative group was set as the 100% control. **(G)** Calories consumption; data are shown as area under curve (AUC) of the experimental period (3 days and 3 nights together). AUC data are presented as % of control; the Cre-negative group was set as the 100% control. **(H)** Cumulative water consumption at the end of the experimental period (3 days). **(I)** Cumulative food intake at the end of the experimental period (3 days). **(J)** Mouse motility (events/20 min) during light and dark period of the three consecutive days and nights was measured by recording motion events with infrared motion sensor. **(K)** Morphology of the gonadal AT from obese mast cell-deficient and -proficient mice was performed with hematoxylin/eosin staining; representative images are shown. Student’s *t*-test **(A–E)** and ANCOVA **(F–J)** with animal body weight as covariant were used for statistical evaluation, *n* = 8–10 mice [in **(A–E)**] and *n* = 4 mice/group [in **(F–J)**]. Data are expressed as means ± SEM.

Crossing of *Mcpt5-Cre* transgenic mice (19) to the *R-DTA* line (27) results in profound deficiency for connective tissue mast cells, the subset of mast cells populating most tissues, including AT, due to selective suicidal expression of diphtheria toxin A in *Mcpt5-Cre^+^R-DTA^+^* animals. Lack of connective tissue mast cells is reflected by absence of IgE-mediated anaphylaxis, whereas the numbers of other major immune cell types are not affected (28). We assessed the involvement of mast cells in diet-induced obesity-related metabolic dysregulation. First, a group of mast cell-deficient and mast cell-proficient littermate control mice was followed on standard diet for >15 weeks. Under these conditions, mast cell-deficient mice displayed no differences with regards to body weight, AT and liver weight, glucose tolerance, and further metabolic parameters, e.g., blood cholesterol, blood triglycerides, or blood insulin, as compared to controls (data not shown). We, then, performed a detailed analysis of mice in the course of HFD-induced obesity. In contrast to *Kit^W-sh/W-sh^* mice (12), but similarly to the *Cpa3^Cre+^* mice (16), *Mcpt5-Cre^+^R-DTA^+^* mast cell-deficient and -proficient (*Cre*-negative *R-DTA^+^*) mice that were fed a HFD did not display any significant differences in body weight (Figure 1A), body weight gain (Figure 1B), glucose tolerance (Figure 1C), and insulin resistance (Figure 1D). Thus, mast cell-deficiency does not affect systemic metabolic dysregulation and development of insulin resistance due to obesity. In accordance with the body weight data, the weights of the sAT, gAT, liver, muscle (quadriceps), and pancreas were not affected by mast cell-deficiency in diet-induced obesity (Figure 2A). Moreover, no differences in fasting blood cholesterol (Figure 2B) and triglyceride levels (Figure 2C) or fasting plasma insulin levels (Figure 2D) were found between obese mast cell-deficient and -proficient mice. Furthermore, mast cell deficiency did not affect adiponectin levels (Figure 2E). We subjected obese mast cell-deficient and -proficient mice to CLAMS analysis and observed no alterations in RER (Figure 2F), calories consumption (Figure 2G), water and food intake (Figures 2H,I), and motility (Figure 2J) between *Cre*-negative and *Cre^+^* mice. Additionally, histological analysis of the AT did not display any differences between obese mast cell-deficient and -proficient mice (Figure 2K), with regards to AT morphology or adipocyte size. Together, mast cells are dispensable in obesity-related metabolic dysregulation and insulin resistance development.

### Mast Cell Deficiency Does Not Affect Obesity-Related AT Inflammation

**Figure 3:**
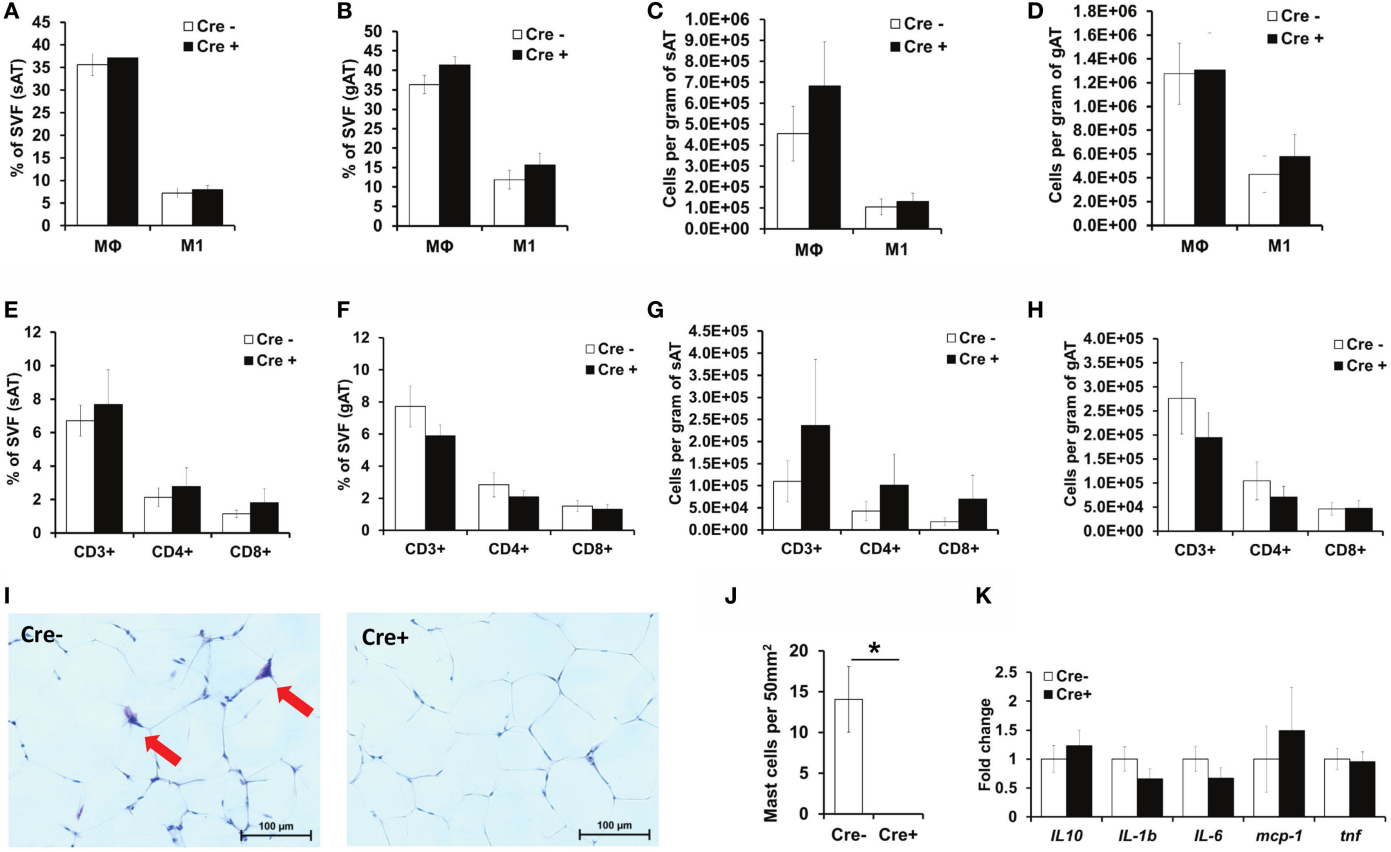
**No difference in AT inflammation between obese mast cell-deficient and -proficient mice**. **(A–H)** The stromal vascular fraction (SVF) of subcutaneous (sAT) and gonadal (gAT) adipose tissue from obese mast cell-deficient and -proficient mice was analyzed by flow cytometry for the accumulation of total macrophages (Mφs) and pro-inflammatory M1-polarized macrophages. The presence of CD3^+^ T lymphocytes and of T helper CD4^+^ and cytotoxic CD8^+^ T cells was analyzed. Student’s *t-*test was used for statistical analysis; data are expressed as means ± SEM (*n* = at least 8 mice/group). **(A,B)** Percentual analysis of macrophage accumulation in the sAT **(A)** and gAT **(B)**. **(C,D)** Number of macrophages per gram of sAT **(C)** and gAT **(D)**. **(E,F)** Percentual evaluation of T cell accumulation in the sAT **(E)** and gAT **(F)**. **(G,H)** Number of T cells per gram of sAT **(G)** and gAT **(H)**. **(I,J)** Representative images (Giemsa staining) for the presence of mast cells in the gAT of obese mast cell-deficient and -proficient mice **(I)**. Mast cell deletion was evaluated **(J)**; *n* = at least 8 mice/group. Mast cells were not present in the AT of *Mcpt5-Cre^+^R-DTA^+^* mice. *p < 0.05. **(K)** Quantitative PCR analysis of inflammatory gene expression in the gAT of obese mast cell-deficient and -proficient mice. Data are shown relative to the mast cell-proficient mouse group; 18S RNA was used for normalization. Mann–Whitney *U* test was used for statistical analysis (*n* = 8 mice/group).

As mast cells have been previously proposed to contribute to a variety of immune responses (8–10) and especially to obesity-related AT inflammation (12), we next evaluated inflammatory cell accumulation in the AT. Consistent with the findings in mast cell-deficient *Cpa3^Cre+^* mice (16), flow cytometric analysis did not reveal any differences in the accumulation of total Mφs or pro-inflammatory M1-polarized CD11c^+^ Mφs in the sAT or the gAT between obese *Mcpt5-Cre^+^R-DTA*^+^ mast cell-deficient and obese *Cre*-negative *R-DTA^+^* control mice (Figures 3A–D). No difference in Mφ accumulation was observed when data were expressed as the percentage of SVF (Figures 3A,B) or when data were expressed as cell numbers per gram of tissue (Figures 3C,D). Similarly, mast cell deficiency did not affect accumulation of total T cell numbers (CD3^+^ cells), CD4^+^ T helper cell numbers, or CD8^+^ cytotoxic T cell numbers in the obese sAT or gAT (Figures 3E–H). Histological analysis of Giemsa-stained sections confirmed the virtual absence of mast cells in gAT of *Mcpt5-Cre^+^R-DTA^+^* mice (Figures 3I,J). Quantitative PCR analysis of the gAT of obese mast cell-deficient and -proficient mice did not reveal any difference in the expression of cytokines IL-10, IL-1β, IL-6, and TNF and of the chemokine MCP-1 and thus in the inflammatory environment of the AT (Figure 3K). In conclusion, mast cells do not regulate AT inflammation in the course of diet-induced obesity.

### Mast Cell Deficiency Does Not Affect Obesity-Related Liver Steatosis and Inflammation

**Figure 4:**
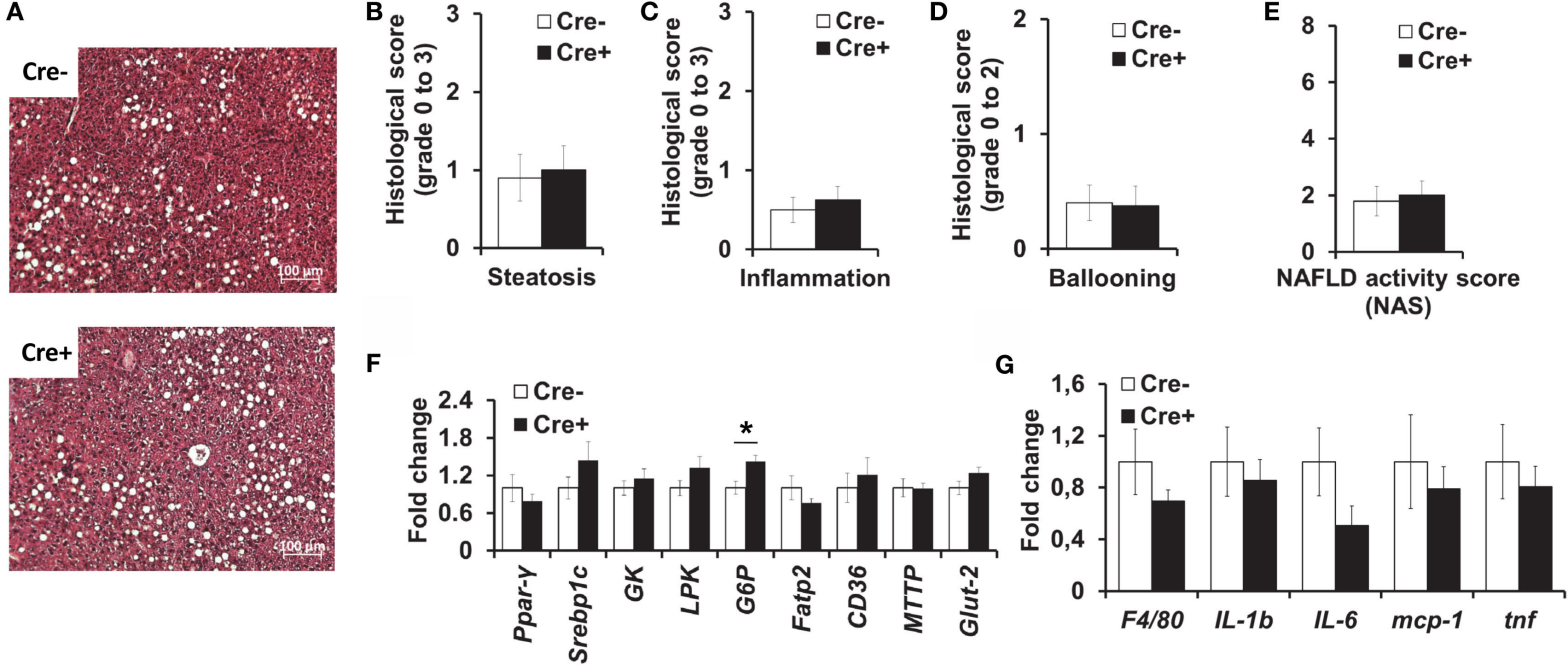
**No difference in liver inflammation and metabolism between obese mast cell-deficient and -proficient mice**. **(A–E)** Liver hematoxylin/eosin-stained paraffin sections from obese mast cell-deficient and -proficient mice were analyzed histologically and evaluated for NAFLD activity score (NAS), consisting of steatosis, lobular inflammation, and hepatocellular ballooning; three sample images (400× power field) per animal were scored (*n* = 8–10 mice). Student’s *t*-test was used for statistical analysis; data are expressed as means ± SEM. **(A)** Representative images of the liver from obese mast cell-deficient and -proficient mice. **(B)** Liver steatosis was scored on a scale from 0 to 3. **(C)** Liver lobular inflammation was scored on a scale from 0 to 3. **(D)** Hepatocellular ballooning was scored on a scale from 0 to 2. **(E)** NAFLD activity score (NAS) represents the combination of steatosis, inflammation, and ballooning score on a scale from 0 to 8. **(F,G)** Quantitative PCR analysis from the livers of obese mast cell-deficient and -proficient mice was performed. Data are shown relative to the mast cell-proficient mouse group; 18S RNA was used for normalization. Non-parametric Mann–Whitney *U* test was used for statistical analysis; data are expressed as means ± SEM (*n* = 8–10 mice). **(F)** Quantitative PCR analysis of metabolic gene expression in the liver; *p < 0.05. **(G)** Quantitative PCR analysis of inflammatory gene expression in the liver.

We also performed histological analysis of the livers of obese mast cell-deficient and -proficient mice. Hepatic steatosis (Figures 4A,B), as well as lobular inflammation (Figure 4C), hepatocellular ballooning (Figure 4D), and overall NAS (Figure 4E) did not differ between the two groups. Moreover, quantitative PCR did not display any differences in the expression of a series of factors involved in hepatic metabolism (Figure 4F), including lipogenesis [peroxisome proliferator-activated receptor gamma (PPARγ), sterol regulatory element-binding protein 1C (Srebp1c)], glycolysis [liver-type pyruvate kinase (LPK), glucokinase (GK)], the transport of fatty acids [fatty acid transport protein 2 (FATP2)], and triglycerides [CD36, microsomal triglyceride transfer protein (MTTP)] and glucose uptake by cells [glucose transporter 2 (Glut-2)], due to mast cell deficiency. The only significant difference between the two groups was in the expression of the gluconeogenic gene glucose-6-phosphatase (G6P, Figure 4F), which was slightly increased in mast cell-deficient mice, as compared to the control group. Further, we analyzed the inflammatory milieu of the liver of obese mast cell-deficient and -proficient mice and found no differences in the expression of F4/80 (as a surrogate marker for the presence of Mφs and Kupffer cells) or of the cytokines IL-1β, IL-6, and TNF and of the chemokine MCP-1 (Figure 4G). Therefore, mast cell deficiency does not contribute to obesity-associated hepatic steatosis and metabolic dysregulation.

## Discussion

It was previously described that the AT of lean and obese mice differ substantially with regards to mast cell numbers (14, 29, 30). In addition, the obesity-related increase of mast cell numbers is more prominent in gAT (29), thereby suggesting that mast cells might participate in obesity-related AT inflammation and metabolic dysregulation (12). The proposed effects of mast cells in obesity, however, were inferred from experiments in *Kit* mutant mast cell-deficient mouse strains *Kit^W/W-v^* and *Kit^W-sh/W-sh^* (12, 13, 18), which displayed significantly lower weight gain and an improved obesity-related metabolic and inflammatory phenotype as compared to control mice. This effect was attributed to the lack of mast cell-derived IFN-γ and IL-6 (12). However, Gutierrez et al. not only demonstrated that absence of mast cells has no effect on AT inflammation and metabolic dysregulation but also showed that the protection of *Kit* mutant mice against the effects of hypercaloric diet results rather from effects of reduced *Kit* expression than from mast cell deficiency (16). We used, here, a mast cell deficiency model that is based on a fundamentally different principle than that of the Cre-MASTER (*Cpa^Cre/+^*) mice employed by Gutierrez et al., however, our data clearly support the latter study.

In diet-induced obesity with mast cell-proficient and -deficient mice, we analyzed various metabolic parameters and we addressed also the inflammatory status of sAT, gAT, and the liver. We did not observe any substantial differences in obesity and obesity-related dysregulation due to mast cell deficiency. The only statistically significant difference was the slightly increased expression of the mRNA of the gluconeogenic factor glucose-6-phosphatase in the livers of mast cell-deficient mice. If anything, slightly enhanced expression of a gluconeogenic factor in mast cell-deficient mice would rather contribute to worse insulin sensitivity in these mice. However, as this was the only difference between the two groups and was not prominent (1.4-fold increase in mRNA levels), we do not consider it a relevant difference.

Our analysis included a series of metabolic parameters and metabolic tests in mast cell-deficient and -proficient mice in the course of obesity; our findings demonstrate that mast cells are dispensable in the pathogenesis of obesity and obesity-related metabolic dysfunction (e.g., glucose intolerance and insulin resistance). Furthermore, we demonstrate, here, that Mφ accumulation and polarization toward the pro-inflammatory M1 population in the obese AT is not affected by the presence or absence of mast cells. In agreement with Gutierrez et al. (16), we conclude that mast cells are also dispensable for the development of obesity-related AT inflammation. Further experimental studies should address the exact crosstalk between mast cells and other immune cells in the obese AT.

Of note, the discrepancy between *Kit* mutant mice and novel models of mast cell deficiency with unperturbed *Kit* function emerging in the analyses of diet-induced metabolic dysregulation is an addition to the already long list of cases in which key findings in *Kit* mutant mice were not reproduced in novel mast cell-deficient mouse strains with normal *Kit* function (11, 17, 20, 28, 31–37). Collectively, overwhelming evidence accumulated over the past few years that *Kit* mutant mice are unreliable as models of mast cell deficiency even if observed phenotypes can be reversed by reconstitution with *in vitro*-differentiated mast cells.

## Author Contributions

JC designed and performed experiments, analyzed and interpreted data, and wrote the manuscript; AC designed and performed experiments and analyzed and interpreted data; K-JC designed experiments and interpreted data; MP performed experiments; DV provided essential experimental tools; AR co-designed the project, analyzed and interpreted data, and edited the manuscript; and TC co-designed the project and wrote the manuscript.

## Conflict of Interest Statement

The authors declare that the research was conducted in the absence of any commercial or financial relationships that could be construed as a potential conflict of interest. The reviewer AF and handling Editor declared their shared affiliation, and the handling Editor states that the process nevertheless met the standards of a fair and objective review.
